# Addressing the affordability gap of novel cancer treatments in developing countries

**DOI:** 10.1371/journal.pdig.0000488

**Published:** 2024-05-01

**Authors:** Gavin Miyasato, Chitrang Shah, Todd Gorsuch, Ramnath Venkateswaran, Vamsi Chandra Kasivajjala, Mohit Misra

**Affiliations:** 1 Mango Sciences, Inc., Boston, Massachusetts, United States of America; 2 Mango Sciences, Inc., Bengaluru, Karnataka, India; Healthtech Advisory, SINGAPORE

Global pharmaceutical companies are creating life-changing treatments but face challenges in maximizing access to these medications, particularly in developing countries. Getting patients to start and stay on treatment remain unsolved issues [1].

The problem spans the entire treatment paradigm, starting at physicians’ willingness to prescribe a high-cost medication and carrying through to patients’ ability to continue paying for the medication. The problem is particularly acute in developing countries due to the low penetration of medical insurance, which leads to high out-of-pocket (OOP) costs. For example, in India, affordability is a major barrier in the private immuno-oncology (IO) market where most patients carry high OOP payments. It is estimated that 98% of patients in India cannot afford IO therapy when recommended by their physician [2]. Based on epidemiological calculations, there may be over 600,000 incident cancer patients annually in India that could be taking these targeted therapies but do not due to affordability challenges [3]. The situation is not unique to India, however, as this problem is observed in most other developing countries in Southeast Asia and Africa where OOP costs are high.

Our on-the-ground research into this issue in India shed light on their presentation in the clinical setting. The patient journey is first impacted by the doctor’s willingness to prescribe the medicine. Novel cancer therapies can have relatively high non-response rates and have not been extensively tested in Indian populations [4]. The adoption of immune checkpoint inhibitors, as an example, is guided by Western data and there is a dearth of published real-world experience in the Indian population, making it difficult to justify their cost and risks [5]. Doctors want to avoid the financial toxicity for patients and their families created by prescribing an expensive drug, let alone one that may not work [6]. Our interviews with leading oncologists corroborated this, as doctors have stated that they perform a basic cost-benefit analysis for each patient that informs their decision to prescribe an expensive, novel treatment or a cheaper generic. Currently, physicians are most concerned about patient financial toxicity and tend to prescribe older, less expensive, and often less effective medications.

Patients’ willingness to buy and ability to pay for the medicine remain a challenge. In India, the cost of novel cancer therapies routinely exceeds $18,000 per year with an estimated 63% of cancer patients facing catastrophic healthcare expenditures [7–8]. Popular IO drug, Keytruda, for example, retails for $82,000 per year [9]. Health insurance penetration in India is growing but is still very low–retail health insurance only covers 3.2% of the population and with the average annual household income of $3,356, most of the innovative medicines are out of reach for most Indians [10]. Moreover, most patients who start novel treatments drop off therapy after exhaustion of funds, thus lowering the duration on therapy and potential for positive treatment outcomes.

To solve these access and affordability challenges in India and other similarly high OOP markets, a multi-stakeholder approach must simultaneously address the various aspects of the problem. Most importantly, patient voices must be heard to develop a program that meets their needs. In addition, healthcare providers must be engaged to understand the treatment decision making process and deploy the solution, pharma companies must be partnered with to ensure a cohesive access strategy, and lenders must be onboarded to confirm the availability of financing options.

Accordingly, Mango Sciences has developed a proof-of-concept of the model, developed by working with 100+ specialty cancer centres and pharma companies, for the OOP market in India. Our three-pronged approach involves:

1. Leveraging AI-powered analytics to predict outcomes for cancer patients and recommend the right treatment for the right patient.
2. Providing patients with value-based agreements tied to the effectiveness of their drug. If the drug works, the patient pays the full amount. If not, a warranty rebate is provided based on the value received by the patient. To enable this, retrospective analysis of electronic medical records is used to measure treatment response in the real-world setting. The results of the analysis determine the maximum rebates that can be paid to patients while keeping the program sustainable.
3. Creating innovative finance offerings focused on “care now, pay later” concepts that provide low or no-interest loans to patients. These specialized lending products provide patients with the ability to pay over time and overcome the prescription abandonment and adherence challenges. We have seen that these loan programs alone have not been successful but believe their combination with data-driven prescribing and value-based agreements make for a compelling patient opportunity.

To illustrate the potential benefit to patients, doctors, and payers, we use an example of Keytruda for the treatment of lung cancer. First and foremost, patients receive the benefit of a warranty program, ongoing support, and a loan option. Meanwhile, treating physicians have the freedom to prescribe the most effective medication with a reduced concern for patient financial toxicity. From the payer perspective, assuming the treatment of 100 cancer patients, a value-based care model tied to progression-free survival provides a savings of ~20%. In addition, the deferred payment model with zero interest allows the payer to treat 3.7X the number of patients each year with the same cash outlay. Quantifying the program impact is straightforward through measurements of patient volumes, physician prescribing patterns, and payer reimbursements.

While the current prototype has been deployed in the private sector, there is potential for a “lift and shift” to government payers in low- and middle-income countries as they seek to improve coverage of these precision oncology products for the wider population.

In summary, the oncology market in India and other developing countries face a large affordability gap that limits doctors’ willingness to prescribe high-cost, novel medicines and inhibits patients’ willingness and ability to pay. Future solutions to solve this problem will focus on a combination of emerging global concepts such as real-world evidence, value-based care, and innovative lending products. By thinking creatively, we can bring together the entire ecosystem to change the paradigm of cancer care for patients in developing countries.
